# Characterizing sexual histories of women before formal sex-work in south India from a cross-sectional survey: implications for HIV/STI prevention

**DOI:** 10.1186/1471-2458-12-829

**Published:** 2012-09-28

**Authors:** Sharmistha Mishra, Satyanarayana Ramanaik, James F Blanchard, Shiva Halli, Stephen Moses, T Raghavendra, Parinita Bhattacharjee, Rob Lorway, Marissa Becker

**Affiliations:** 1 Department of Infectious Diseases Epidemiology, Imperial College London, London, UK; 2 Department of Medicine, St. Michael’s Hospital, University of Toronto, Toronto, Canada; 3 Karnataka Health Promotion Trust, Bangalore, India; 4 Center for Global Public Health, University of Manitoba, Winnipeg, Canada

**Keywords:** Sex-work, HIV prevention, Sexual life-course, India, Devadasis

## Abstract

**Background:**

Interventions designed to prevent HIV and STIs in female sex-workers (FSWs) reach women after they formally enter the sex-trade. We aimed to characterize the pattern of sexual behaviour among FSWs from first-sex to when they identify as sex-workers (transition period) in a region with traditional (historically characterized by dedication into sex-work at first-sex) and non-traditional forms of sex-work.

**Methods:**

We conducted a cross-sectional survey of 246 traditional and 765 non-traditional FSWs across three districts in Karnataka, India. We performed univariate and multivariate logistic regression to profile FSWs most likely to engage in a commercial first-sex before identifying as a sex-worker. Sexual life-course patterns were distinguished using univariate and multivariate linear regression based on key events associated with length of transition period.

**Results:**

Overall, 266 FSWs experienced a commercial first-sex, of whom 45.9% (95% CI: 38.2,53.7) continued a long-term relationship with the first partner. In adjusted analysis, traditional FSWs were more likely to experience a commercial first-sex (AOR 52.5, 95% CI: 27.4,100.7). The average transition time was 8.8 years (SD 3.9), but there was considerable variability between respondents. Among women who experienced a commercial first-sex, a slower transition was independently associated with non-traditional sex-work, the presence of long-term partnerships during the transition period, and ongoing partnerships at time of entry into sex-work. In the absence of a commercial first-sex, a faster transition was associated with traditional sex-work and the dissolution of long-term partnerships, while a slower transition was associated with the presence of long-term partnerships and widowhood. Only 18.5% (95% CI: 12.7,26.2) and 47.3% (95% CI: 32.7,62.3) of women reported ‘always’ condom use with their long-term and occasional partners during the transition period, respectively.

**Conclusions:**

FSWs identify as sex-workers several years after becoming sexually active, even when the first-sex is commercial in nature. Long-term partnerships are common after a commercial first-sex, and are associated with a delay in formally entering the sex-trade. The findings call for a better understanding of HIV/STI risk before FSWs identify as sex-workers, and an adaptive programme to reach this period of vulnerability.

## Background

The vulnerabilities that place female sex-workers (FSWs) at risk of HIV and sexually transmitted infections (STIs) during formal sex-work are well characterized in India [1,2]. Interventions designed to address vulnerabilities have increased protective behaviours and decreased HIV/STI prevalence among FSWs [3-6]. In the state of Karnataka, HIV prevalence in FSWs fell from 19.6% to 16.4% between 2004 and 2009 [3]. Existing programmes for FSWs are designed to reach women after they identify as a sex-worker [6,7]. The proposed AIDS Risk Reduction Model suggests that an individual’s perception of risk leads to self-labelling and protective behaviours [8]. As a result, intervention impact may be limited if women engage in high-risk behaviour before they identify as a sex-worker (i.e. formally enter the sex-trade). In this paper, we define the transition period as the time from first-sex to self-declared (or formal) entry into sex-work. Behavioural surveillance suggests that this transition may take up to 20 years in Karnataka [2,9]. However, there has been little research into the sexual behaviour of an FSW after she becomes sexually active but before she considers herself a sex-worker.

Evidence suggests that FSWs in northern Karnataka may be at risk of acquiring HIV during the transition period. Representative cross-sectional surveys report that 21% of practicing FSWs are infected with HIV before their second year in sex-work [10]. In a prospective cohort study of FSWs, 2.6% of women died of AIDS before their second year in practice, and HIV-attributable mortality did not increase with a longer duration of sex-work [11]. Both studies were conducted after extensive mapping of high-risk groups in India based on established methodologies for hidden and vulnerable populations. In both studies, >90% of participants had accessed local HIV/STI prevention services at time of enrolment or survey [10,11]. While these findings do not distinguish between HIV exposures during the early years of formal sex-work and the transition period, the early AIDS-attributable mortality in particular suggests a risky transition period (given our understanding of average time from HIV seroconversion and AIDS) [12,13].

Potential vulnerabilities during the transition period include established risk factors for HIV outside of formal sex-work [14,15]: number of sexual partners, a partner’s number of other partners, and inconsistent condom-use. Furthermore, the different ways in which women enter the industry could impact on the length and characteristics of the transition period. In northern Karnataka, transactional sex is practiced in the context of traditional (*Devadasi*) and non-traditional sex-work [1]. The *Devadasi* tradition dates back to the 6^th^ century AD in India [16,17]. Adolescent girls were ‘dedicated’ by their families to temples, where *Devadasis* worked as dancers and courtesans and were not allowed to marry [16,17]. Over time, the tradition became one of home-based sex-work, but important elements of what it means to identify as a ‘*Devadasi’* remain. The classification of ‘*Devadasi*’ is passed down from mother to one of her daughters [16,17]. An adolescent girl officially becomes a *Devadasi* during a dedication ceremony that takes place soon after her menarche [16,17]. As part of this ceremony, a *Devadasi*’s first sexual partner is a client (often an older man) who pays for her initiation [16,17]. In this region, a higher HIV prevalence among clients is correlated with older age [9,18]. Because the first sexual encounter occurs at the time of dedication into this ritualized form of sex-work [16,17], *Devadasis* should not experience a true transition period. In contrast, non-traditional FSWs may enter the industry several years after first-sex, particularly because reasons for sex-work in Karnataka include economic hardship following spousal abandonment or widowhood among previously married women [19]. Furthermore, there is variability in HIV/STI risk across sex-work typologies: there is some evidence to suggest that *Devadasis* have a 2-fold higher HIV prevalence than non-traditional FSWs [2].

A prospective, longitudinal cohort of sexually active women would be the best study design to outline the sexual life-course of FSWs before they enter sex-work, but this type of study is resource-intensive and may not reach women who are most likely to enter the sex-trade. Recently, Boileau and colleagues demonstrated a novel approach to examining sexual life-histories using a cross-sectional survey of adult women in Malawi [20]. The study categorized life-course trajectories based on a sequence of sexual and partnership events in a woman’s life in relation to current HIV prevalence [20]. Although causal pathways along a trajectory could not be inferred from the retrospective data, the study highlighted important insights into the sequence of life-course events associated with HIV risk [20].

We undertook a cross-sectional study of FSWs in northern Karnataka to describe the sexual behaviour of FSWs during the transition period, and to profile FSWs most likely to experience a commercial first-sex. We then quantified the sources of variability in transition time based on key events in the women’s lives [20] before they formally entered sex-work [17]. We used this information to categorize FSWs according to the main sources of variability in transition length by sex-work typology.

## Methods

### Study setting and population

The study took place in three northern districts in Karnataka, India: Belgaum, Bagalkot, and Bijapur. Targeted HIV/STI interventions for FSWs have been implemented in northern Karnataka since 2004, and fall under the remit of a large-scale HIV prevention programme (the National AIDS Control Organization [6] and *Avahan*, the India AIDS initiative of the Bill & Melinda Gates Foundation [21]). Before and during implementation, the programme conducted two extensive mapping exercises to enumerate high-risk populations in the region [6,7,9]. An estimated 20,493 women practice sex work across the three districts [9], of whom 38.3% are *Devadasis*[9]. FSWs are registered with the program at first contact with a peer-educator or outreach worker who actively seek and identify FSWs on an ongoing basis. As of January 2011, 97% of enumerated FSWs had been registered with the local programme [9]. For this study, the sample frame included 11,028 registered FSWs who were both currently engaged in sex work and had reported (to the programme) that they started sex work in the last five years. The median duration of formal sex-work in the region is 7 years [2,22], and the study was restricted to ‘new’ entrants to limit recall bias as much as possible.

We used conventional cluster sampling with probability proportional to size of the enumerated FSW population in each village. A total of 1,500 FSWs were sampled. If FSWs were not at their place of residence during the first study visit, two repeat visits were made as well as contact through community researchers (peers) to arrange interviews. Trained interviewers obtained written informed consent and conducted face-to-face interviews with a structured questionnaire in the local language (*Kannada*). At this stage, interviewers independently confirmed study eligibility: FSWs who had received money or gifts in exchange for sex in the past year and reported starting sex-work after December 2005 were included in the study. The surveys were conducted between January and June 2011. The questionnaire collected data on socio-demographic characteristics, sexual behaviour, and sexual partnerships. Prior to study commencement, the questionnaire underwent pilot testing with FSWs (community researchers).

### Definitions and statistical analysis

We defined first-sex as commercial if it involved any of the following: (a) the first sexual partner was considered to be a ‘client’ by the respondent [as opposed ‘boyfriend’, ‘husband’, ‘neighbour’]; (b) first-sex was associated with a dedication ceremony; or (c) first-sex included the receipt of money or gifts in exchange for sex (and differentiated from dowry). The migratory profile of FSWs was divided into three categories [22]: (a) migrant (FSWs who practiced sex-work outside their home district for more than two weeks per year, in addition to sex-work at home); (b) mobile (FSWs who engaged in sex-work outside their village or city of residence for less than two weeks per year, in addition to sex-work at home); (c) local (FSWs who practiced sex-work only in their village or city of residence).

The ‘self-reported’ start of sex-work was obtained by asking FSWs to recall the year (and if possible, the month), and the age when they ‘first entered sex-work or started taking clients, where sex-work is defined as the receipt of money or gifts in exchange for sex’. Both questions (year/month and age) are standardized across surveys in India [2-4] and elsewhere [23-25], and are currently used in sex-worker programmes [6] to define ‘duration in sex-work’. To calculate the transition period, we used information on age at entry because age at first-sex was felt to be more reliable than year of first-sex.

Participants were asked to provide structured details on each regular partnership during the transition period. The questionnaire defined a regular sexual partner as any man with whom the respondent had sex with on more than one occasion. The reported duration of regular partnerships was greater than six months, and none of the regular partnerships were classified as ‘regular clients’. We therefore use the term ‘long-term partnership’ in place of ‘regular partnership’ in this paper.

Condom use during long-term partnerships was categorized as ‘always’ if the participant answered ‘always’ separately for each partner, and ‘not always’ otherwise. Based on pilot testing, the question was posed in reference to the total duration of each long-term partnership. For occasional partners, FSWs were asked to recall if condoms were ‘always’, ‘sometimes’, or ‘never’ used without specifying each partner, and dichotomized for the analysis as ‘always’ or ‘not always’.

Characteristics of study participants, first-sex, and sexual behaviour during the transition period are described across sex-work typology: *Devadasi* and non-*Devadasi*. We used univariate and multivariate logistic regression to profile FSWs most likely to have engaged in a commercial first-sex before formally entering the sex-trade. We examined patterns of sexual behaviour from cross-sectional data, as previously described [20], using the following variables: commercial first-sex, long-term partnership during the transition period, and partnership status at time of self-reported entry into sex-work. These key events are similar to major transitions among sexually active women [20], but were modified in the context of traditional sex-work [16,17]. We used univariate and multivariate linear regression to quantify the variability in length of transition period explained by key events, and stratified results by type of first-sex. To categorize sexual life-histories, we used sequential divisions along the key events if: (a) the events were associated with variability in the transition period; and (b) the sample size within categories was greater than 10 (to achieve a reasonable sample size within cells of categorical variables that allows for meaningful interpretation). All analyses were adjusted for within-cluster homogeneity to account for the sampling design, and performed in Stata 11 (StataCorp, USA). Institutional review boards at the University of Manitoba, Canada and St. John’s Medical College, Bangalore, India provided ethical approval.

## Results

### Study population

Of the 1,500 FSWs sampled, 1, 214 (80.9%) could be reached and were invited to participate. In total, 1,182 (97.4%) consented to the interview, and among them, 1,011 met eligibility criteria. Compared to respondents, women who declined to participate or could not be reached (N=318) were more likely to have formally engaged in sex work for >3 years (OR 1.4, 95% CI: 1.1, 1.7), and were more likely to live in Bijapur (OR 1.2, 95% CI: 1.1, 1.3). There was no difference in the migration status, typology, or age of respondents versus non-respondents, although these characteristics were limited to the programme registration data.

The study consisted of 333 (32.9%), 507 (50.2%), and 171 (16.9%) FSWs who started sex work in 2006-2007, 2008-2009, and 2010-2011 respectively. The median age of participants was 22 years (range, 15 to 32). Table 1 highlights the socio-demographic profile and characteristics of first-sex by typology. *Devadasi* women were more likely to move across districts and states for sex-work, although the majority of FSWs restricted their practice to their residential village or city (Table 1). The mean age at first-sex was 15.3 years, with no significant difference between the FSW typologies. More than a third of FSWs became sexually active before age 15 (Table 1). Non-*Devadasis* were more likely to become sexually active within a year of menarche (Table 1).

**Table 1:** Profile of study participants and the characteristics of first-sex

	**Traditional sex-work (Devadasi) N=246% (95%, CI)**	**Non-Devadasi, N=765%, (95%, CI)**	***p***
**Rural residence**	61.4 (42.0,77.7)	69.3 (57.2,79.2)	0.4
**Current age (years)**			
<20	19.1 (13.5,26.3)	4.2 (2.8,6.1)	
20-24	45.9 (38.0,54.1)	19.5 (16.2,23.2)	
≥25	35.0 (26.0,45.1)	76.3 (71.9,80.3)	<0.001
**Able to read andwrite**	26.4 (19.5,34.7)	30.5 (26.6,34.6)	0.4
**Migration status**			
Local	52.9 (42.1,63.3)	68.2 (62.4,73.6)	
Mobile	13.8 (8.3,22.2)	27.3 (22.4,32.8)	
Migrant	33.3 (24.4,43.6)	4.4 (3.0,6.6)	<0.001
Ever married	5.7 (2.1,7.7)	92.2 (89.5,94.2)	<0.001
**Age at first-sex (years)**			
10-14	32.9 (26.5,40.1)	38.0 (32.3,44.0)	
15-19	63.0 (56.0,69.5)	59.7 (53.7,65.4)	
20-25	4.1 (2.3,7.1)	2.4 (1.4, 4.0)	0.2
**Fraction sexually active ≤1 year after menarche**	17.1 (112.8,22.4)	27.5 (22.6,33.0)	0.004
**First-sex took place outsidevillage/city of residence**	11.8 (8.1,16.8)	8.9 (7.0,11.3)	0.001
**Commercial first-sex**	82.9 (76.6,87.8)	8.1 (6.2,10.5)	<0.001
**Condoms used at first-sex**	26.4 (19.5,34.8)	2.1 (1.3,3.5)	<0.001

### Characterizing first-sex

All *Devadasis* had participated in a dedication ceremony, but only 69.9% (95% CI: 61.4,77.2) did so at first-sex. The remainder entered the *Devadasi* tradition at a later point in time. Of note, 17.9% (95% CI: 12.3,25.3) of *Devadasis* participated in more than one dedication ceremony during the transition period.

In 26.3% (95% CI: 21.5,31.8) of participants, the first-sex act could be classified as commercial in nature (Table 1). Of these 266 women, only 13.9% (95% CI: 9.2,20.5) considered the commercial first-sex as their point of entry into the sex-trade. A commercial first-sex was significantly more common among *Devadasis* (Tables 1, 2). After adjusting for sex-work typology, migrant FSWs (Adjusted Odds Ratio [AOR] 0.39, 95% CI: 0.17,0.89) who formed a long-term partnership with their first partner (AOR 0.12, 95% CI: 0.07,0.20) were less likely to experience a commercial first-sex (Table 2). A later sexual debut was independently associated with a commercial first-sex (AOR 3.4, 95% CI: 1.3,8.9).

**Table 2:** Profile of female sex workers who experienced a commercial first-sex compared against FSWs without a commercial first sex (N=1,011)

	**First sex commercial sex act (N=266)**					
	**N**	**Column % (95%, CI)**	**Crude OR (95%, CI)**	***p***	Adjusted OR* 95%, CI)	***p***
**Able to read andwrite (N=298)**	74	27.8 (21.5,35.2)	0.89 (0.61,1.3)	0.6	---	---
**Rural residence (N=681)**	167	62.8 (47.0,76.2)	0.76 (0.44,1.3)	0.3	---	*---*
**Sex-work typology**						
Traditional (*Devadasi*, N=246)	204	76.7 (67.7,83.8)	55.1 (32.5,93.3)	<0.001	52.5 (27.4,100.7)	<0.001
Non-traditional (N=765)	62	23.3 (16.2,32.3)	Ref		Ref	
**Migration status**						
Local (N=652)	154	57.9 (48.8,66.5)	Ref		Ref	
Mobile (N=243)	46	17.3 (11.7,24.8)	0.76 (0.44,1.3)	0.3	0.86 (0.36,1.5)	0.6
Migrant (N=116)	66	24.8 (17.7,33.7)	4.2 (2.7,6.8)	<0.001	0.39 (0.17,0.89)	0.03
**First sexual partner becamea long-term partner**						
Yes (N=808)	122	45.9 (38.3,53.6)	0.07 (0.05,0.11)	<0.001	0.12 (0.07,0.20)	<0.001
No (N=203)	144	54.1. (46.4,61.7)	Ref		Ref	
**Age at first-sex (years)**						
10-14 (N=371)	86	32.3 (26.7,38.5)	Ref		Ref	
15-19 (N=611)	167	62.8 (56.6,68.6)	1.2 (0.89,1.7)	0.2	1.0 (0.63,1.7)	0.9
20-25 (N=28)	13	4.9 (3.0,8.0)	2.9 (1.2,6.7)	0.02	3.4 (1.3,8.9)	0.01

OR (odds ratio). *Adjusted for sex-work typology, migration status, first sexual partner, and age at first-sex.

### Sexual behaviour and partnerships during the transition period

Table 3 depicts the number and type of partnerships formed during the transition period. Overall, 38.6% of *Devadasis* and 49.4% of non-*Devadasis* reported at least two partners during the transition period (Table 3). At least three sexual partners were reported by 23.5% (95% CI: 19.8,27.7) of FSWs. Multiple long-term partnerships were more common among non-*Devadasis* (Table 3). Half of women without a commercial first-sex (49.5%, 95% CI: 44.6, 54.4) reported at least two sexual partners during the transition period (median 2, range 2 to 10). Reported ‘always’ condom use was low overall, but lower with long-term partners than with occasional partners (Table 3).

**Table 3:** Sexual behaviour and partnerships prior to formal sex-work

	**Traditional sex-work (*Devadasi***) **N=246% (95%, CI)**	**Non-*Devadasi*, N=765% (95%, CI)**	***p***
**Age of first sexualpartner**			
<20	11.4 (7.4,17.2)	21.2 (18.3,24.4)	
20-40	83.3 (77.0,88.2)	78.2 (74.9,81.1)	
40-60	3.7 (1.8,7.2)	0.3 (0,1.1)	
Do not know/recall	1.6 (0.6,4.6)	0.4 (0.1,1.2)	<0.001
**Relationship with first-sexual partner**			
Boyfriend	13.8 (9.8,19.2)	10.0 (7.3,12.7)	
Husband	1.6 (0.5,5.4)	80.7 (77.8,83.2)	
Neighbour	9.4 (6.0,14.4)	5.4 (3.7,7.7)	
Client/Dedication partner	75.2 (67.0,81.9)	4.3 (2.9,6.3)	<0.001
**First sexual partner becamea long-term partner**	48.4 (40.8,56.0)	90.1 (87.6,92.1)	<0.001
**Total number of sexualpartners during transition period(including first-sex)**			
1	61.4(54.9,67.5)	50.6(45.7,55.5)	
2	21.1(16.1,27.2)	23.9(20.3,28.0)	
≥3	17.5(12.8,23.4)	25.5(21.2,30.4)	<0.03
**Total number of long-termpartners during transition period(including first-sex)**			
0	30.9(24.4,38.3)	4.4(3.1,6.4)	
1	39.0(33.1,45.3)	51.0(46.0,55.9)	
2	19.5(14.4,25.9)	30.5(26.8,34.4)	
≥3	10.6(6.9,15.8)	14.1(11.3,17.5)	<0.001
**Total number of occasionalpartners during transition period(including first-sex)**			
0	82.5(76.4,87.3)	81.8(76.8,86.0)	
1	11.0(7.7,15.4)	8.6(6.6,11.2)	
2	3.3(1.7,6.1)	5.8(3.9,8.4)	
≥3	3.3(1.3,7.8)	3.8(2.0,7.1)	0.4
Condom use with long-termpartners during transition period*			
Always	14.8 (7.5,27.1)	19.6 (12.8,28.9)	
Not always (sometimes/never)	85.2 (72.9,92.5)	80.4 (71.1,87.2)	0.4
Condom use with occasionalpartners during transition period**			
Always	34.9 (19.2,54.7)	51.1 (34.1,67.8)	
Not always (sometimes/never)	65.1 (45.3,80.8)	48.9 (32.2,65.9)	0.2
**Partnership status at self-reportedentry into sex-work**			
Ongoing long-term partnership	30.1(23.2,38.0)	47.5 (41.4,53.6)	
Widowed	1.6 (0.7,4.2)	19.9(17.0,23.1)	
Partnership dissolved	34.4 (31.6,42.0)	31.8(27.0,37.0)	
No long-term partner	30.9 (24.4,38.3)	0.92(0.4,2.1)	<0.001

*Among participants who reported at least 1 regular partner during the transition period. **For participants who reported at least 1 occasional partner during the transition period. *p*-value from chi-squared tests of association.

### Characterizing sexual life-histories

**Figure 1:**
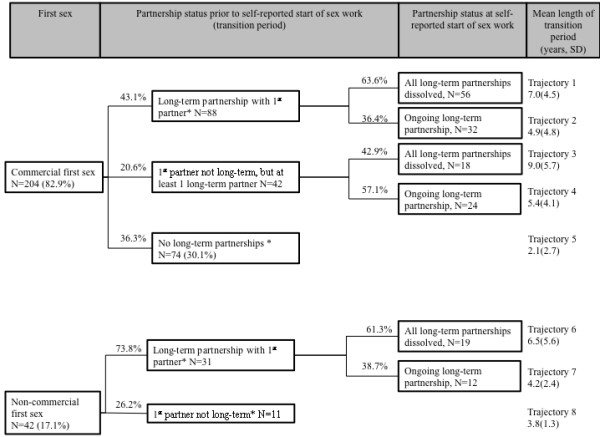
**Sexual life-histories of traditionalsex-workers (*Devadasis*).** Percentages within boxes refer to the total sample (N=256), while percentages along the connecting lines refer to the sample size in the preceding box. *Includes women who formed long-term partnerships with someone other than their first sexual partner; partnership status at start of sex-work was not further divided among women who did not form long-term partnerships with their first-sexual partner because of small sample size. The mean duration of each trajectory is shown in years with the standard deviation (SD).

**Figure 2:**
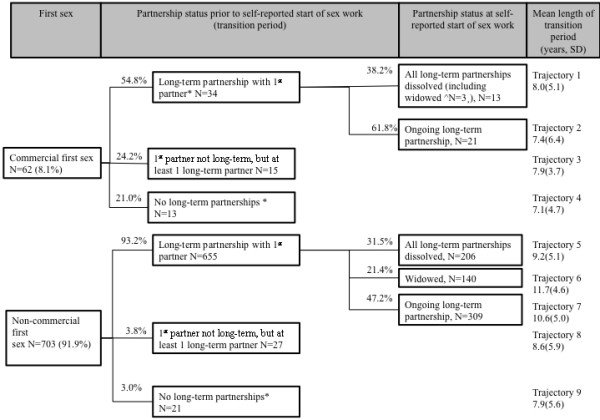
**Sexual life-histories of non-*Devadasis*.** Percentages within boxes refer to the total sample (N=765), while percentages along the connecting lines refer to the sample size in the preceding box. *Partnership status at start of sex-work was not further divided in the group of FSWs who did not form long-term partnerships with their first sexual partner because of small sample size (<10). The mean duration of each trajectory is shown in years (y) with the standard deviation (SD).

The average length of the transition period was 8.8 years (SD 3.9), but considerable variability was observed. Table 4 summarizes the association between key events and length of transition period, stratified by type of first-sex. Using this information, the sequence of events in the transition period among *Devadasis* and non-*Devadasis* is outlined in Figures 1 and 2, respectively [20].

**Table 4:** Association between sex-work typology and partnership events with length of transition period, by type of first-sex

	**First-sex = commercial**				**First-sex≠commercial**			
	** Univariate**		** Multivariate**		** Univariate**		** Multivariate**	
	**Slope (95%, CI)**	***p***	**Slope (95%, CI)**	***p***	**Slope (95%, CI)**	***p***	**Slope (95%, CI)**	***p***
**Sex-work typology**								
Traditional (*Devadasi*)	-2.7 (-4.4,-0.96)	0.003	-1.7 (-3.7,-0.3)	0.04	-5.1 (-6.7,3.5)	<0.001	-4.1 (-5.9,-2.3)	<0.001
Non-traditional	Ref		Ref		Ref		Ref	
**Partnerships during the transitionperiod**								
1^st^ partner became a long-term partner	Ref		Ref		Ref		Ref	
1^st^ partner did not become a long-term partner, but had at least 1 long-term partner	0.56 (-1.1,2.2)	0.5	0.79 (-0.82,2.4)	0.3	-2.5 (-4.2,-0.95)	0.002	-1.3 (-2.9,0.23)	0.09
**Partnership status at self-reportedstart of sex work**								
Ongoing long-term partnership	Ref		Ref		Ref		Ref	
All long-term partnerships dissolved	1.5 (-0.09,3.1)	0.07	1.8 (0.3,3.3)	0.02	1.5 (0.45,2.6)	0.005	-1.3 (-2.4,-0.22)	0.02
Widowed	2.6 (-0.3,5.4)	0.08	1.7 (-1.3,4.6)	0.3	-1.5 (-2.6,-0.31)	0.01	1.2 (0.16,2.3)	0.02
No long-term partners (during transition period)	-3.7 (-5.0,-2.4)	<0.001	-2.6 (-4.0,-1.2)	<0.001	-7.5 (-10.1,-5.0)	<0.001	-5.1 (-9.1,-1.2)	0.01
R^2^			0.47				0.51	

Transition period refers to the time between first-sex and self-reported start of sex-work. The slope represents the change in transition time (in years) compared to the reference group.

Of the 266 FSWs who experienced a commercial first-sex, 45.9% (95% CI: 38.2, 53.7) continued a long-term partnership with their first sexual partner. None of these women classified this first long-term partner as a ‘regular client’. An average of 4.9 years (SD 4.8) had elapsed before women in this subgroup referred to themselves as sex-workers. In the absence of a commercial first-sex, the mean transition time was 9.9 years (SD 5.2).

For women who reported a commercial first-sex, the dissolution of long-term partnerships was independently associated with a longer transition, while the absence of any long-term partnerships was associated with an earlier transition, after adjusting for typology (Table 4). In the absence of a commercial first-sex, some of the variability in transition time was explained by the following (Table 4): the presence of a long-term partnership (slower transition), widowhood (slower transition), dissolution of long-term partnerships (faster transition), and traditional sex-work (faster transition). However, only 47% and 51% of the variability in transition length could be explained by the available information (Table 4).

*Devadasis* without long-term partnerships reported the shortest transition (mean 2.1 years, Figure 1). The longest transition was reported by widowed, non-traditional FSWs who did not experience a commercial first-sex (mean 11.7 years, Figure 2). After adjusting for first-sex, partnership status during the transition period, and partnership status at entry into sex-work, *Devadasis* transitioned into sex work 2.7 years (95% CI:1.3,4.2) earlier than non-traditional sex workers (p<0.001).

Despite the traditional restriction on marriage, 48.4% of *Devadasis* formed a long-term partnership with their first sexual partner (Table 3), and 5.7% were married (Table 1). By the time they formally entered the sex-trade, a third of *Devadasi* were still in an ongoing long-term partnership (Figure 1, Table 3). The majority of non-traditional FSWs formed long-term partnerships with their first-sexual partner (representing marriage in >90%, Figure 2, Table 3). Half of these partnerships had dissolved or resulted in widowhood by the time non-*Devadasis* started sex-work.

## Discussion

Targeted HIV prevention programmes are designed to address HIV/STI risk in FSWs after they identify as sex-workers. We documented a considerable gap between a commercial first-sex and formal entry into the sex-trade, and significant variability in the transition time in a region with traditional and non-traditional forms of sex-work. A quarter of FSWs experienced a commercial first-sex, but on average identified themselves as sex-workers 4.9 years later. After a commercial first-sex, half of women entered into a long-term partnership with the first client. This study was not designed to measure HIV/STI risk during the transition period. However, our findings suggest that an absence of self-recognition of HIV/STI risk, the commercial nature of first-sex and long-term partnerships with the first client, and consequently, risky behaviours of long-term male partners, could work together in concert to place FSWs at risk of HIV/STIs during the transition period. As a result, the findings from this study suggest important implications for HIV/STI prevention interventions, and raise important questions for further research.

There was considerable variability in the transition time reported by FSWs. The variability stems from differences in when a woman perceives herself to be a sex-worker. Across typologies, the presence or absence of long-term partnerships was an important source of this variability. Women who formed at least one long-term partner after a commercial first-sex reported a longer transition time. The findings suggest an important role for long-term partnerships with respect to whether (or when) a woman identifies herself as a sex-worker following a transactional first-sex act. In India, as elsewhere, sex-work is defined as the exchange of money or gifts for sex [26], and is self-reported by a woman using the questions employed in this study [2,23,27]. But the transactional nature of such partnerships may be clandestine [28], sporadic, or range across a wide spectrum with respect to type and number of partners [28,29]. Sex-worker interventions are often restricted to the end of the spectrum where client numbers are in excess of several hundred per year – as in the case of India [2].

Most women who acknowledged the receipt of money/gifts in exchange for sex during their first sexual encounter did not equate this event with entry into the sex-trade, despite having been provided the same definition when asked about formal entry into sex-work. Currently, programmes and studies ask participants about duration in sex-work on the basis of an ‘exchange of money or gifts for sex’ [2,27]. Our findings suggest that FSWs may not report their commercial first-sex act when asked this standardized question. Therefore, programmes may under-estimate the duration of transactional sex in a woman’s life and fail to reach women until years after their commercial first-sex. None of our study participants would have been reached with existing sex-worker programmes during their transition period. While HIV prevention messages exist for the general population, interventions targeted to FSWs are vastly different. The latter involves an intensive condom-based and STI treatment programme that includes community mobilization, peer-education and outreach, and structural interventions to address sexual violence, substance abuse, and personal finance [6,7,27,30,31]. Therefore, timing of entry into formal sex-work (or self-identification as a sex-worker), and the fraction of HIV/STI acquisition that occurs before a woman identifies as a sex-worker could have important implications for her own health and for HIV transmission in the wider community [32]. After adjusting for long-term partnership status and age at first-sex, the profile of FSWs most likely to have experienced a commercial first-sex includes *Devadasis* who do not migrate for sex-work. The individual-level association between temporary migration by FSWs and HIV prevalence remains controversial and complex [2,33], partly due to limited data on changes in sexual behaviours between home and destination. Our findings suggest that potential risk during the transition period could add another layer of complexity when comparing HIV prevalence across migration status. FSWs who are at higher-risk of a commercial first-sex (and potentially HIV acquisition during the transition period) are less likely to migrate for sex-work, such that reliable comparisons of HIV prevalence between migrant, mobile, and local FSWs may need to account for potential HIV acquisition prior to formal sex-work.

The findings from this study underscore the importance of characterizing the HIV/STI risk and sexual behaviour of long-term partners of women who eventually identify as sex-workers. This is particularly relevant for women who form long-term partnerships with their first client. None of the FSWs classified the commercial first-sex partner as a ‘regular client’ after forming a long-term partnership with him. While our study does not provide sufficient data on the extent to which this long-term partnership was transactional in nature, the high HIV prevalence among clients in northern Karnataka suggests that FSWs who form long-term partnerships with them (prior to formal entry into sex-work) are at risk of HIV. By 2009, the HIV prevalence among clients of FSWs in Bagalkot district was 18.2% [9]. Men who pay for sex in India often have multiple other partnerships [34-36]. Reported condom use at last sex in long-term relationships between clients and self-identified FSWs ranges between 22% and 40% [3,37], compared with over 80% in commercial partnerships [3,37]. Our findings suggest that the same low level of condom-use in long-term partnerships exists at a time when women do not consider themselves to be sex-workers. Less than 20% of FSWs reported ‘always’ using condoms with their long-term partners during the transition period. Additional study is required to examine the difference in risk posed to partners of clients who do and do not eventually enter the sex-trade. While our findings suggest that vulnerabilities likely exist during the transition period among FSWs who experience a commercial first-sex, a better understanding of male partners is required to delineate the exact nature and timing of the vulnerabilities.

Contrary to our previous understanding of the *Devadasi* tradition [1,16,17], we found that up to a third of *Devadasis* were inducted after their first-sex, and 17.9% experienced repeated ‘dedication ceremonies’. Not surprisingly, traditional FSWs experienced a faster transition than non-*Devadasis*, but the average transition time for *Devadasis* ranged from 2.1 to 9.0 years across the sexual life-histories depicted in Figure 1. Recent work suggests that this traditional form of sex-work in Karnataka may be evolving, influenced partly by the introduction and enforcement of laws prohibiting dedication ceremonies [38]. Even less is known about the pathway into sex-work among non-traditional FSWs [19]. In this study, 49.4% of non-traditional FSWs reported multiple partnerships, with a median of 2 sexual partners (long-term and occasional) during the transition period. General population surveys conducted in this region suggest that only 1.2-2.4% of women report more than one sexual partner in their lifetime [11,39]. Our study does not allow for a direct comparison of sexual histories among non-*Devadasi* women in the region who did and did not formally enter sex-work. Future research is needed to understand pathways into sex-work among non-*Devadasi* women, and whether factors such as number of sexual partners are associated with formal entry into sex-work.

To our knowledge, this is the first study that attempts to characterize the sexual histories of FSWs before they identify as sex-workers. However, our findings were limited by the cross-sectional study design. Categories of sexual life-histories were limited to a quantitative search for sources of variability in transition time. Causal pathways could not be inferred. For example, we cannot infer a predictive relationship between commercial first-sex and eventual formal transition into sex-work, and this remains an important and unanswered question. Nor could we disentangle or quantify the interactions between the suggested sources of HIV/STI risk during the transition period. The study sample was restricted to FSWs registered with the local HIV/STI intervention programme. The programme has achieved a high coverage of enumerated FSWs, and extensive mapping has been an integral part of the programme [7]. Nonetheless, we may have missed FSWs not yet identified by mapping or by the programme. Social desirability bias could affect our findings. For example, women may not have reported a transactional history with their first-sex act because dedications into the *Devadasi* tradition are prohibited by law. Furthermore, accuracy of the reported age at which specific events take place is limited by recall bias. We attempted to limit recall bias by restricting inclusion to new entrants. The questions used to calculate the transition period are widely used to define the ‘start of sex-work’ and ‘duration of sex-work’ [2,23], and therefore comparable to the wider literature and to programmes. After stratifying by commercial first-sex, the final regression models only explained 47% and 51% of observed variability in transition time, suggesting that unmeasured factors exist which influence when a woman perceives herself to be a sex-worker. The variability in responses (and therefore, variability in the transition time) points to the importance of understanding the switch in a woman’s perception of whether she is or is not a sex-worker (apart from the ‘exchange of money or gifts for sex’). Further research is needed, including qualitative studies and a biological assessment of HIV/STI risk during the transition period. A qualitative study to examine the transition period and the transactional nature of long-term partnerships among FSWs is currently underway. Further research using a prospective longitudinal study design to measure HIV/STI incidence during the transition and early period of formal sex-work is also under development.

## Conclusions

We documented potential high-risk sexual behaviour of FSWs during the transition period, and profiled FSWs most likely to experience a commercial first-sex. While this study was not designed to quantify HIV/STI risk during the transition period, the findings raise an important public health question: should current HIV prevention programmes be adapted to address sexual behaviour before women formally enter the sex-trade? FSWs identify as sex-workers several years after becoming sexually active, even when the first-sex is commercial in nature. A long-term partnership with a client is common during the transition period, and is associated with a delay in formally entering the sex-trade. Identifying and reaching this group of women will be challenging, and targeted interventions could be difficult to operationalize, due to the already hidden nature and stigma of formal sex-work. Yet HIV prevention programmes for FSWs are dependent on a woman’s self-identification as a sex-worker; it is only at this point that women have access to, or are accessed by, HIV prevention interventions. Hence, our findings suggest a need for HIV prevention programmes to reach not only young sex-workers, but young women. Prevention programmes targeted to FSWs in Karnataka have demonstrated success in reducing risky behaviours, STIs, and HIV [3,4]. This study calls for further research to quantify the risk of STIs and HIV before women identify as sex workers, and the need to expand programmes to reach women during the transition period.

## Competing interests

The authors declare that they have no competing interests (financial or non-financial).

## Authors’ contributions

MB conceived of the study, participated in its design, coordinated implementation of the study and drafted the manuscript. SM participated in the design and implementation of the study, conducted the analysis and drafted the manuscript. RS, SH, TR and PB were involved in the implementation of the study as well as contributing to review and finalization of the manuscript. JB, SMoses and RL were involved in the study design and contributed to the review and finalization of the manuscript. All authors read and approved the final manuscript.

## Pre-publication history

The pre-publication history for this paper can be accessed here:

http://www.biomedcentral.com/1471-2458/12/829/prepub
